# Alcoholic Cardiomyopathy Mortality in the United States (1999-2024): Trends, Disparities, and Opportunities for Prevention

**DOI:** 10.7759/cureus.117430

**Published:** 2026-10-04

**Authors:** Anuj Timshina, Anukul Subedi, Prabin Duwadee, Paribesh Gyawali, Laxman Wagle

**Affiliations:** 1 Internal Medicine, Ascension Saint Agnes Hospital, Baltimore, USA; 2 Emergency Department, Dhading District Hospital, Dhading, NPL

**Keywords:** alcoholic cardiomyopathy, cardiovascular prevention, cardiovascular-related mortality, health inequalities, heart failure

## Abstract

Aim: This study aimed to characterize long-term US mortality from alcoholic cardiomyopathy (ACM), compare its trajectory with other major cardiomyopathy subtypes, and identify demographic and geographic disparities relevant to cardiovascular prevention.

Methods: We conducted a serial cross-sectional analysis of Centers for Disease Control and Prevention Wide-ranging Online Data for Epidemiologic Research (CDC WONDER) underlying-cause-of-death data from 1999 to 2024. Cardiomyopathy deaths were identified using ICD-10 codes I42.0, I42.1, I42.2, I42.5, I42.6, I42.8, and I42.9. Annual age-adjusted mortality rates (AAMRs) were assessed overall and by sex; age-specific crude death rates (CDRs) were recalculated for ages 15-44, 45-64, and 65-84 years. ACM mortality was also examined by race and state.

Results: From 1999 to 2024, AAMRs declined for dilated cardiomyopathy (1.965 to 0.716 per 100,000; -63.6%), unspecified cardiomyopathy (7.196 to 3.155; -56.2%), and other hypertrophic cardiomyopathy (0.450 to 0.228; -49.2%). ACM declined less, from 0.199 to 0.144 (-27.7%), with an increase during the early 2020s. Male ACM AAMR declined from 0.408 to 0.209 but remained nearly fourfold the female rate in 2024 (0.054). Mortality was concentrated among middle-aged and older males; American Indian or Alaska Native individuals had the highest population-based ACM mortality, and Alaska had the highest pooled state AAMR in 2018-2024.

Conclusion: ACM showed a smaller long-term mortality reduction than several major cardiomyopathy subtypes and persistent demographic and geographic disparities. Integrating cardiovascular prevention and heart failure care with recognition and treatment of harmful alcohol use may help reduce this potentially modifiable burden.

## Introduction

Cardiomyopathies are a heterogeneous group of myocardial disorders characterized by structural and functional abnormalities that may lead to heart failure, arrhythmias, and sudden cardiac death [1,2]. Alcoholic cardiomyopathy (ACM) is an acquired form of dilated cardiomyopathy associated with prolonged heavy alcohol exposure in the absence of another sufficient cause [3]. Its pathogenesis is multifactorial and includes direct ethanol toxicity, oxidative stress, mitochondrial dysfunction, and apoptosis [3,4].

The public health burden of excessive alcohol use has increased in the United States. The average annual number of deaths attributable to excessive alcohol use increased by 29.3%, from 137,927 during 2016-2017 to 178,307 during 2020-2021, while the corresponding age-standardized mortality rate increased from 38.1 to 47.6 deaths per 100,000 population [5]. Although these estimates encompass multiple acute and chronic conditions, they provide important context for alcohol-associated cardiovascular disease.

Prior national work reported increasing ACM-attributed mortality from 2010 through 2020 [6]. Whether ACM has followed the broader long-term improvement observed for other cardiomyopathy subtypes, and how its contemporary burden varies across demographic and geographic groups, remains incompletely characterized. We therefore examined US mortality attributed to ACM from 1999 through 2024, compared its trajectory with other major cardiomyopathy subtypes, and evaluated ACM mortality by sex, age, race, and state.

## Materials and methods

Study design and data source

We conducted a serial cross-sectional study of cardiomyopathy-related mortality in the United States from 1999 through 2024 using underlying-cause-of-death data from the Centers for Disease Control and Prevention Wide-Ranging Online Data for Epidemiologic Research (CDC WONDER). Data for 1999-2017 were obtained from the Underlying Cause of Death 1999-2020 database, and data for 2018-2024 were obtained from the Underlying Cause of Death by Single Race 2018-2024 database [7,8]. CDC WONDER compiles mortality information from death certificates filed in the 50 states and the District of Columbia. We included deaths among U.S. residents in which a cardiomyopathy diagnosis of interest was recorded as the underlying cause of death. Because these data are publicly available, de-identified, and aggregate, institutional review board approval and informed consent were not required.

Cardiomyopathy classification

Deaths were identified using International Classification of Diseases, Tenth Revision codes for dilated cardiomyopathy (I42.0), obstructive hypertrophic cardiomyopathy (I42.1), other hypertrophic cardiomyopathy (I42.2), other restrictive cardiomyopathy (I42.5), ACM (I42.6), other cardiomyopathies (I42.8), and cardiomyopathy, unspecified (I42.9). Endomyocardial (eosinophilic) disease (I42.3), endocardial fibroelastosis (I42.4), and cardiomyopathy due to drugs and other external agents (I42.7) were excluded because sparse or suppressed data limited stable longitudinal comparison. ACM was evaluated separately and compared with the included nonalcoholic subtypes.

Mortality measures and sex-stratified analysis

The primary mortality measure was the annual age-adjusted mortality rate (AAMR), expressed as deaths per 100,000 population, with corresponding 95% confidence intervals obtained from CDC WONDER. Age adjustment used the 2000 US standard population. Annual AAMRs were evaluated overall and separately among males and females.

Age-stratified analysis

Age-specific mortality was evaluated using crude death rates (CDRs), separately by sex. CDC WONDER data were extracted in component age categories and aggregated into prespecified groups of 15-44, 45-64, and 65-84 years. Individuals aged ≥85 years and those with age not stated were excluded. Death counts and population denominators were summed across component age categories, and CDRs were recalculated as (total deaths/total population) × 100,000 rather than by averaging component rates.

Race stratification

Race-stratified ACM mortality was evaluated separately for 1999-2017 and 2018-2024 because CDC WONDER racial classification changed between these periods. The earlier dataset categorized individuals as American Indian or Alaska Native, Asian or Pacific Islander, Black or African American, or White, whereas the 2018-2024 Single Race dataset used more granular racial categories. Race-specific mortality measures were described separately by period, and direct comparisons were restricted to sufficiently comparable racial categories.

Geographic stratification

State-specific ACM AAMRs and 95% confidence intervals were obtained for pooled periods 1999-2017 and 2018-2024. Geographic patterns were described by comparing state-specific pooled AAMRs between periods. States with suppressed estimates were excluded from direct comparison. These comparisons were descriptive and do not represent continuous state-specific temporal trends.

Treatment of suppressed values

For aggregated age-specific analyses, some component age-sex strata contained suppressed death counts for which the exact number of deaths was unavailable in CDC WONDER. Because these counts could not be directly ascertained, suppressed values were coded as zero before summation. Because suppressed values represent unknown small numbers of deaths rather than necessarily zero events, this approach may underestimate CDRs, particularly in younger individuals, females, and other low-event strata.

Statistical analysis

Annual and pooled mortality rates were summarized descriptively. Overall and sex-specific temporal patterns were evaluated using annual AAMRs; age-specific patterns were evaluated using recalculated CDRs. Endpoint absolute and relative changes were calculated between 1999 and 2024. No formal temporal trend, between-period, or between-group hypothesis testing was performed; therefore, observed differences are reported descriptively and should not be interpreted as statistically significant. R software (version 4.6.0; R Foundation for Statistical Computing, Vienna, Austria) was used for data visualization and generation of study figures.

## Results

Overall and subtype-specific mortality

Cardiomyopathy mortality generally declined between 1999 and 2024, although the magnitude of change varied by subtype (Table 1; Figure 1). Unspecified cardiomyopathy had the highest AAMR, declining from 7.196 to 3.155 per 100,000 (−56.2%). Dilated cardiomyopathy showed the largest relative decline among major specified subtypes, from 1.965 to 0.716 (−63.6%), and other hypertrophic cardiomyopathy declined from 0.450 to 0.228 (−49.2%). Smaller declines were observed for obstructive hypertrophic cardiomyopathy (0.064 to 0.043; −33.4%) and ACM (0.199 to 0.144; −27.7%). Other restrictive cardiomyopathy remained relatively stable (0.027 to 0.026), whereas other cardiomyopathies increased from a low baseline (0.008 to 0.100).

**Table 1:** Selected overall and sex-specific age-adjusted mortality rates by cardiomyopathy subtype, 1999 and 2024 AAMRs are expressed as deaths per 100,000 population. Relative change represents the percentage change in AAMR from 1999 to 2024. The large relative increase observed for other cardiomyopathies reflects the low baseline AAMR in 1999 and should be interpreted cautiously. Abbreviations: AAMR, age-adjusted mortality rate; ACM, alcoholic cardiomyopathy

Subtype	Population	1999 AAMR	2024 AAMR	Relative change
Dilated cardiomyopathy	Overall	1.965	0.716	−63.6%
Dilated cardiomyopathy	Male	2.846	1.041	−63.4%
Dilated cardiomyopathy	Female	1.266	0.450	−64.4%
Obstructive hypertrophic cardiomyopathy	Overall	0.064	0.043	−33.4%
Other hypertrophic cardiomyopathy	Overall	0.450	0.228	−49.2%
Other restrictive cardiomyopathy	Overall	0.027	0.026	−3.5%
Alcoholic cardiomyopathy	Overall	0.199	0.144	−27.7%
Alcoholic cardiomyopathy	Male	0.408	0.209	−48.8%
Alcoholic cardiomyopathy	Female	0.047	0.054	+15.9%
Other cardiomyopathies	Overall	0.008	0.100	+1220.2%
Cardiomyopathy, unspecified	Overall	7.196	3.155	−56.2%
Cardiomyopathy, unspecified	Male	9.855	4.181	−57.6%
Cardiomyopathy, unspecified	Female	5.334	2.253	−57.8%

**Figure 1:**
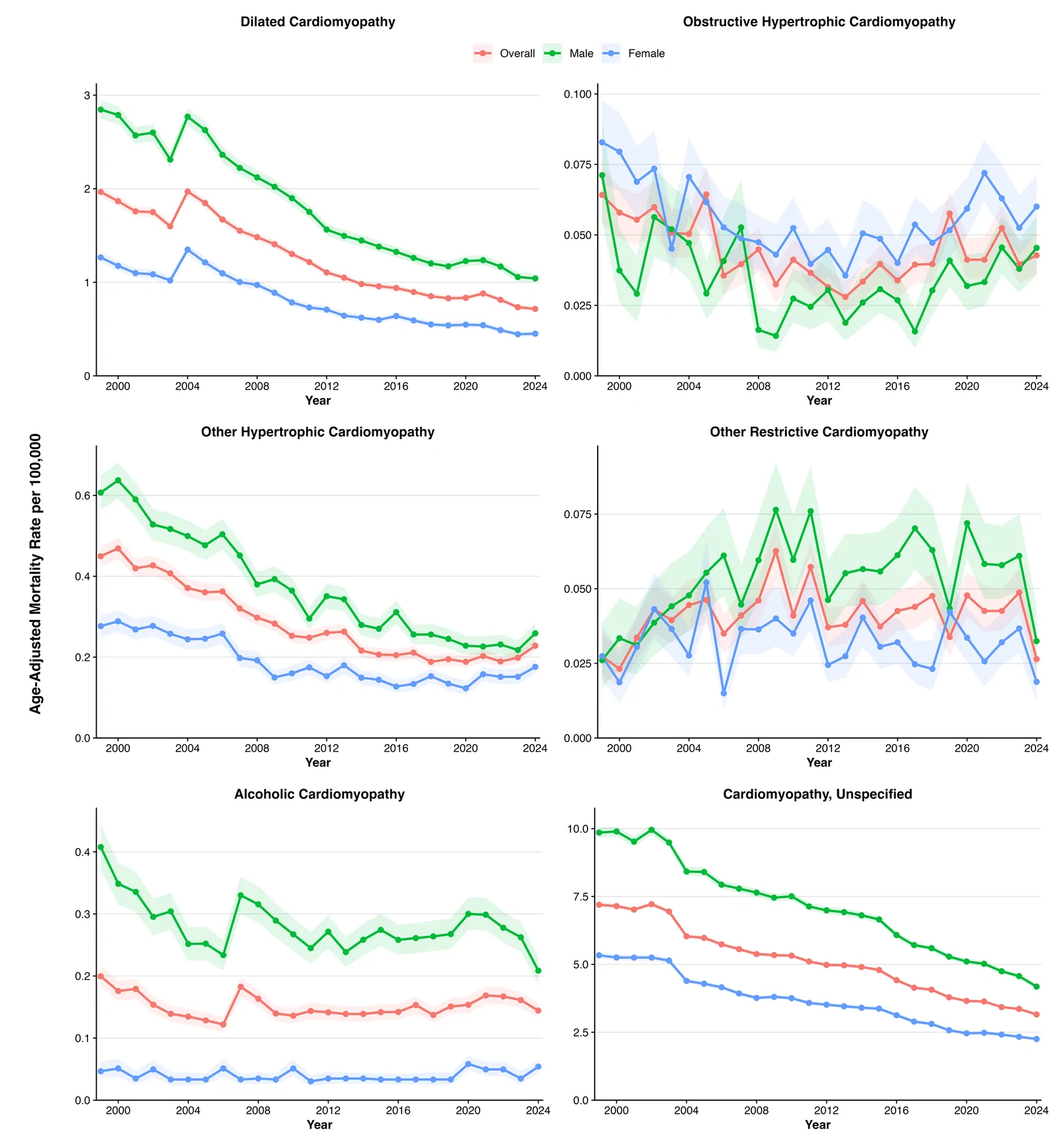
Temporal trends in age-adjusted mortality rates for cardiomyopathy subtypes by sex, 1999–2024 Annual age-adjusted mortality rates (AAMRs) per 100,000 population for dilated cardiomyopathy, obstructive hypertrophic cardiomyopathy, other hypertrophic cardiomyopathy, other restrictive cardiomyopathy, alcoholic cardiomyopathy, and unspecified cardiomyopathy from 1999 through 2024. Trends are presented for the overall population and stratified by sex (male and female). Shaded areas represent 95% confidence intervals. R software (version 4.6.0; R Foundation for Statistical Computing, Vienna, Austria) was used for data visualization and generation of the figure.

Alcoholic cardiomyopathy and sex

ACM showed a smaller endpoint reduction than dilated, unspecified, and other hypertrophic cardiomyopathy and an increase during the early 2020s before declining in 2023-2024 (Figure 1). Male ACM AAMR declined from 0.408 per 100,000 in 1999 to 0.209 in 2024 (−48.8%), whereas the female AAMR changed from 0.047 to 0.054 (+15.9%). Despite the male decline, the 2024 male AAMR remained nearly four times the female rate (Table 1).

Age- and sex-specific mortality

ACM mortality varied substantially by age and sex and was concentrated among middle-aged and older males (Table 2; Figure 2). Among males, CDRs declined from 0.16 to 0.10 per 100,000 among those aged 15-44 years, from 0.89 to 0.47 among those aged 45-64 years, and from 1.03 to 0.57 among those aged 65-84 years between 1999 and 2024. Female CDRs were substantially lower and remained relatively stable among those aged 15-44 and 45-64 years, whereas the rate among females aged 65-84 years increased from 0.09 to 0.11 per 100,000.

**Table 2:** Age- and sex-specific crude death rates for overall and alcoholic cardiomyopathy, 1999 and 2024 CDR indicates crude death rate. Rates are expressed as deaths per 100,000 population. Persons aged ≥85 years and those with age not stated were excluded. For aggregated age-specific analyses, suppressed death counts were coded as zero before summation

Mortality category	Sex	Age, y	1999 CDR	2024 CDR
Overall cardiomyopathy	Male	15–44	2.79	1.66
Overall cardiomyopathy	Male	45–64	13.47	7.27
Overall cardiomyopathy	Male	65–84	58.15	19.92
Overall cardiomyopathy	Female	15–44	1.17	0.67
Overall cardiomyopathy	Female	45–64	5.92	3.10
Overall cardiomyopathy	Female	65–84	30.57	11.27
Alcoholic cardiomyopathy	Male	15–44	0.16	0.10
Alcoholic cardiomyopathy	Male	45–64	0.89	0.47
Alcoholic cardiomyopathy	Male	65–84	1.03	0.57
Alcoholic cardiomyopathy	Female	15–44	0.02	0.03
Alcoholic cardiomyopathy	Female	45–64	0.09	0.09
Alcoholic cardiomyopathy	Female	65–84	0.09	0.11

**Figure 2:**
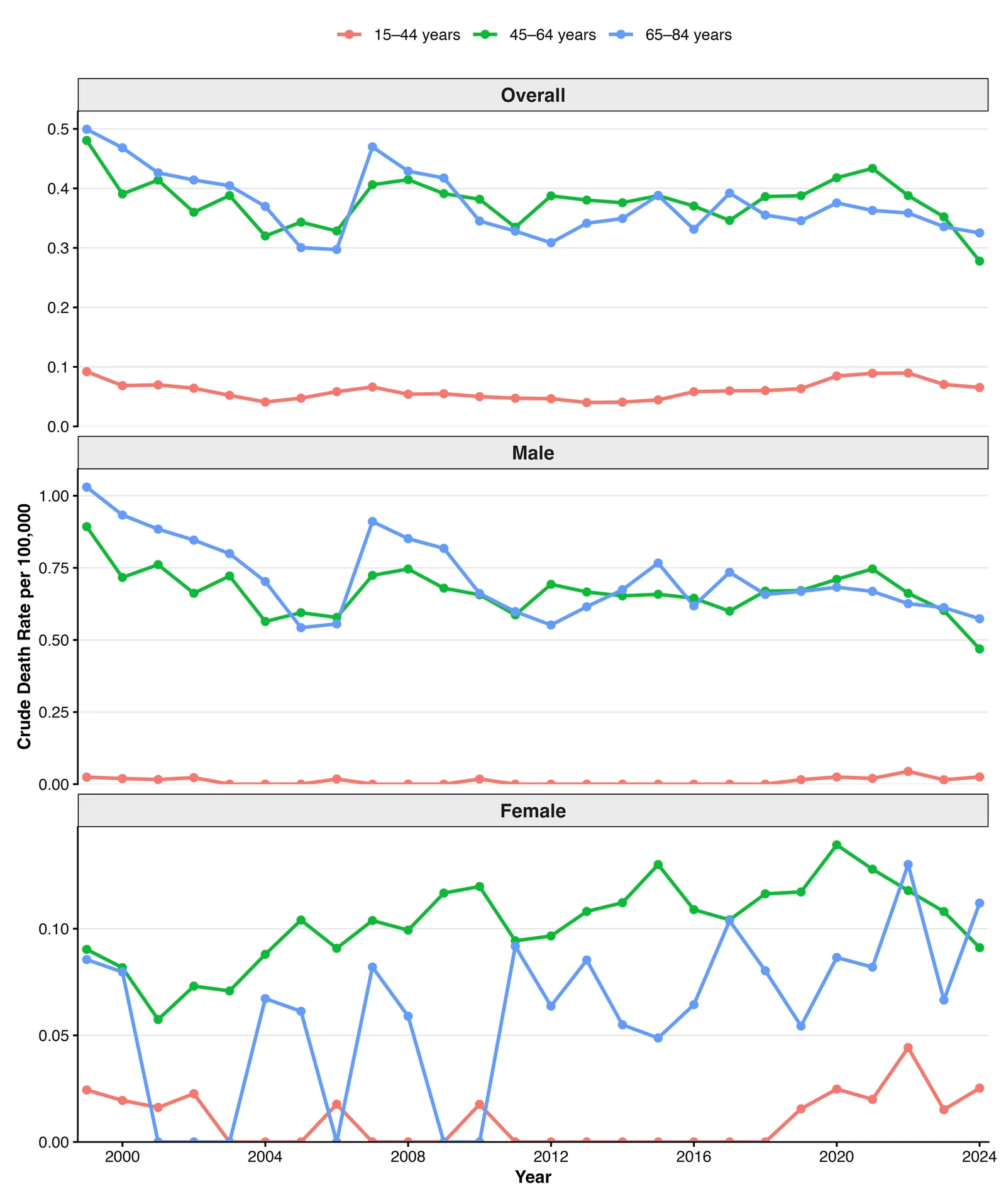
Age- and sex-specific crude death rates for alcoholic cardiomyopathy in the United States, 1999–2024 CDR indicates crude death rate. Rates are expressed as deaths per 100,000 population. Suppressed death counts were coded as zero before aggregation. R software (version 4.6.0; R Foundation for Statistical Computing, Vienna, Austria) was used for data visualization and generation of the figure.

Racial differences

Racial disparities in ACM mortality were observed across both study periods. During 1999-2017, American Indian or Alaska Native individuals had the highest ACM AAMR (0.6 per 100,000), followed by Black or African American (0.3), White (0.2), and Asian or Pacific Islander individuals (0.0). During 2018-2024, American Indian or Alaska Native individuals again had the highest AAMR (0.6 per 100,000) among the racial groups evaluated, compared with 0.2 per 100,000 among both Black or African American and White individuals. White individuals accounted for the largest absolute number of ACM deaths in both periods, with 7,282 deaths (77.9%) during 1999-2017 and 3,306 deaths during 2018-2024.

Geographic variation

State-level ACM mortality demonstrated substantial geographic heterogeneity. Alaska had the highest pooled AAMR during 2018-2024 (1.342 per 100,000), compared with 0.651 per 100,000 during 1999-2017. Higher pooled AAMRs in 2018-2024 than in 1999-2017 were also observed in Utah (0.248 vs. 0.074), Oregon (0.368 vs. 0.222), Tennessee (0.280 vs. 0.179), Iowa (0.290 vs. 0.198), and Washington (0.336 vs. 0.259). Lower pooled AAMRs in 2018-2024 than in 1999-2017 were observed in New Jersey (0.105 vs. 0.214), California (0.170 vs. 0.248), North Carolina (0.137 vs. 0.184), Virginia (0.092 vs. 0.122), and New York (0.115 vs. 0.137). The District of Columbia estimate for 2018-2024 was suppressed, precluding direct comparison between periods (Figure 3).

**Figure 3:**
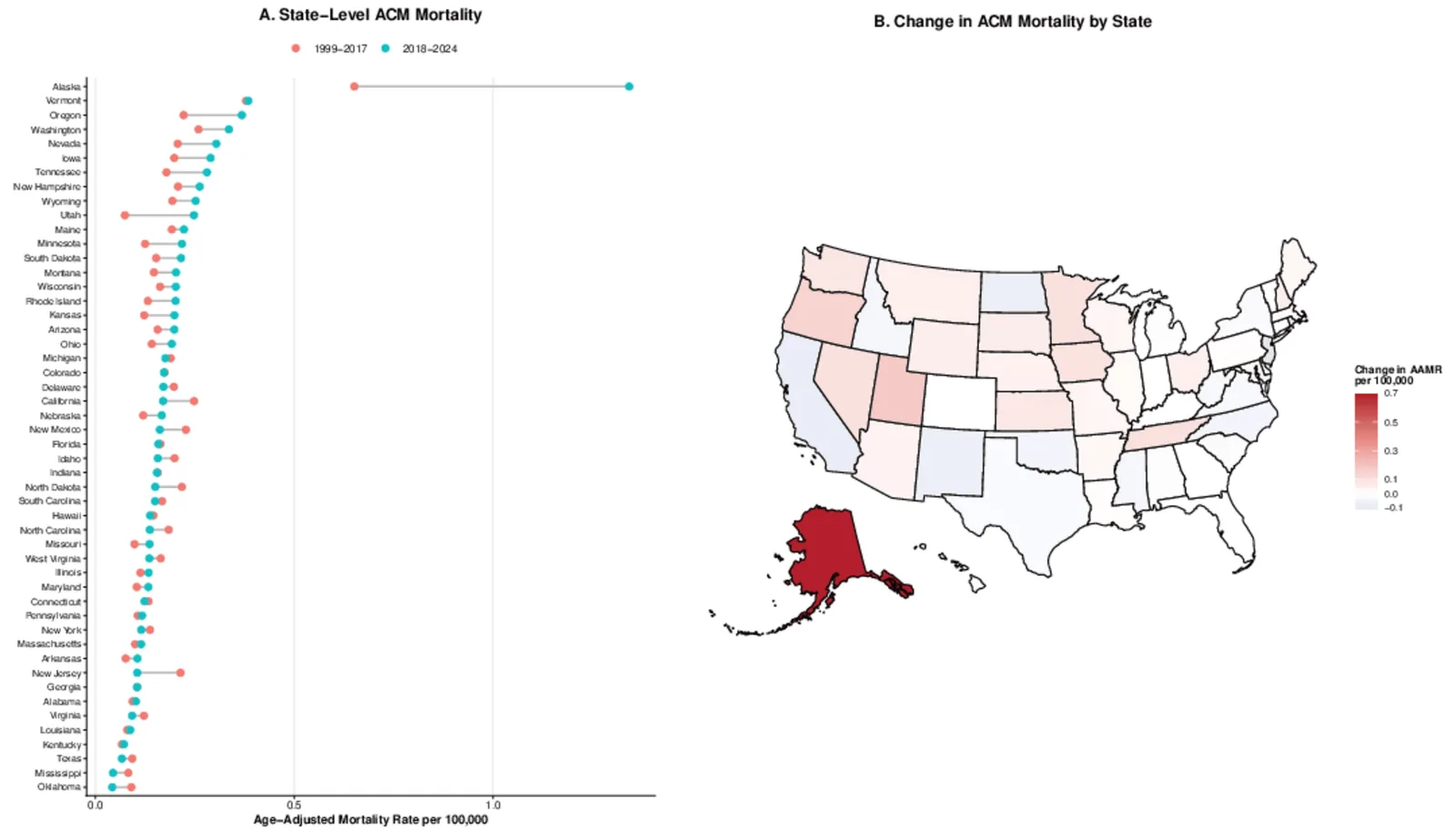
Geographic variation in alcoholic cardiomyopathy mortality in the United States, 1999–2024 (A) State-level pooled age-adjusted mortality rates (AAMRs) for 1999–2017 and 2018–2024; connecting lines indicate changes between periods within each state. (B) Absolute change in state-level AAMR, calculated as the 2018–2024 AAMR minus the 1999–2017 AAMR. States with suppressed estimates were excluded from direct period comparisons. AAMR indicates age-adjusted mortality rate. Abbreviation: ACM, alcoholic cardiomyopathy R software (version 4.6.0; R Foundation for Statistical Computing, Vienna, Austria) was used for data visualization and generation of the figure.

## Discussion

In this nationwide analysis of cardiomyopathy mortality from 1999 through 2024, we observed substantial heterogeneity across cardiomyopathy subtypes. Dilated, unspecified, and other hypertrophic cardiomyopathy showed the largest endpoint reductions in age-adjusted mortality, whereas ACM showed a comparatively smaller decline. ACM was also characterized by a persistent male predominance, concentration of mortality among middle-aged and older males, the highest population-based burden among American Indian or Alaska Native individuals, and substantial geographic variation. These findings suggest that the broad improvement observed for several cardiomyopathy subtypes has not been mirrored to the same extent in ACM.

The decline in dilated cardiomyopathy mortality occurred during a period of substantial advances in the diagnosis, classification, and management of cardiomyopathies [1]. These advances were not directly measured in our study and therefore cannot be assigned as causes of the observed decline. Similarly, the reduction in unspecified cardiomyopathy may partly reflect greater diagnostic specificity and changes in death-certificate coding over time rather than an equivalent reduction in disease incidence.

ACM declined from 0.199 to 0.144 per 100,000, a 27.7% endpoint reduction, and showed an increase during the early 2020s. Prior national work reported increasing ACM-attributed mortality from 2010 through 2020 [6]. Our study extends observation through 2024 and places ACM alongside other cardiomyopathy subtypes. The longer horizon shows that, despite a lower rate in 2024 than in 1999, the magnitude of improvement was smaller than for dilated, unspecified, and other hypertrophic cardiomyopathy. Because no formal comparative trend testing was performed, this should be interpreted as a descriptive difference in trajectories rather than evidence of statistically different trends.

This distinction is clinically relevant because ACM management requires treatment of both heart failure and the underlying alcohol exposure. Guideline-directed heart failure therapy remains central to the treatment of left ventricular systolic dysfunction, while alcohol abstinence is a cornerstone of ACM management; substantial reduction in alcohol consumption has also been associated with recovery of ventricular function in observational cohorts [3,9,10]. Persistent heavy alcohol exposure, relapse, delayed recognition of alcohol use disorder, or limited engagement with addiction treatment could therefore attenuate gains achieved through advances in heart failure therapy. These mechanisms were not available in CDC WONDER and remain plausible explanations rather than demonstrated mediators.

The early-2020s increase occurred during a broader rise in alcohol-associated harm in the United States. Deaths attributable to excessive alcohol use increased substantially between 2016-2017 and 2020-2021, and survey data documented changes in alcohol use and related consequences during the COVID-19 pandemic [5,11]. However, individual alcohol exposure, healthcare utilization, and treatment were unavailable, and the observed concurrence cannot establish causality.

A pronounced sex disparity persisted throughout the study period. Although male ACM mortality declined substantially, the male AAMR remained nearly fourfold the female rate in 2024. Age-specific analyses further localized the burden to middle-aged and older males. This pattern is consistent with prior studies demonstrating a disproportionate burden of alcohol-attributable mortality among men [5,6]. Differences in cumulative alcohol exposure may contribute, along with differences in comorbidity, healthcare utilization, and recognition or treatment of alcohol use disorder. Importantly, the lower observed ACM mortality among women should not be interpreted as lower biological susceptibility to alcohol-related injury; women may experience alcohol-related adverse health effects, including cardiovascular effects, at lower levels of exposure than men [10,12]. Among adults aged 18-25 years, adjusted past-month binge drinking prevalence was higher among women than men during 2021-2023, whereas women had lower prevalence than men during 2017-2019 [13]. These changing exposure patterns may not yet be reflected in ACM mortality, which likely represents the consequences of prolonged cumulative alcohol exposure. The persistent mortality disparity observed in our study nevertheless identifies middle-aged and older men as an important population for prevention and early recognition of alcohol-associated myocardial disease.

Racial disparities were also evident. American Indian or Alaska Native (AI/AN) individuals had the highest ACM AAMR among the racial groups evaluated during 1999-2017 and the highest AAMR among comparable racial groups during 2018-2024. These findings should not be interpreted as biological effects of race. Prior U.S. data similarly demonstrate a disproportionate alcohol-related mortality burden among AI/AN populations, whereas White populations have historically reported a higher prevalence of current alcohol consumption than several other racial and ethnic groups [14,15]. Importantly, racial and ethnic differences in alcohol-attributable mortality persist even after accounting for differences in alcohol consumption, suggesting that alcohol exposure alone does not explain these disparities [14]. Social determinants of health, differences in alcohol consumption patterns, and disparities in access to and utilization of alcohol use disorder treatment may contribute [14,16]. Racial misclassification on death certificates may additionally underestimate mortality among AI/AN populations [17]. White individuals accounted for the largest absolute number of ACM deaths in both periods, emphasizing the distinction between absolute burden and population-based mortality rates. Changes in CDC racial classification limit direct comparison of Asian and Pacific Islander populations across periods.

Marked geographic heterogeneity further suggests that national averages obscure important local differences. Alaska had the highest pooled AAMR during 2018-2024, and several states had higher later-period rates, while others had lower rates. The mechanisms underlying these state-level differences cannot be determined from the present data and may reflect variation in alcohol exposure, population characteristics, socioeconomic and healthcare factors, and death-certification practices. National data showing greater increases in alcohol-induced mortality in rural than urban areas provide additional context but do not directly explain ACM-specific state differences [18]. Because the state analysis compared pooled periods and estimates from smaller states may be unstable, these findings should be considered hypothesis-generating rather than evidence of continuous state-specific trends.

These findings have implications for heart failure prevention and care. Alcohol exposure should be systematically assessed in patients with otherwise unexplained left ventricular systolic dysfunction. Management of ACM should integrate guideline-directed heart failure therapy with evidence-based identification and treatment of unhealthy alcohol use [3,9,10]. The concentration of mortality among middle-aged and older males, the disproportionate population-based burden among American Indian or Alaska Native individuals, and geographic heterogeneity identify populations in which improved prevention, surveillance, and linkage between cardiovascular and addiction care may be particularly relevant.

This study has several strengths, including nationwide coverage, a 26-year observation period extending through 2024, direct comparison of multiple cardiomyopathy subtypes, and detailed demographic and geographic stratification. Several limitations should also be considered. CDC WONDER relies on death certificates and is susceptible to incomplete documentation, diagnostic misclassification, and coding variation; ACM may be particularly underrecognized when alcohol exposure is not documented, or its contribution is uncertain. The analysis was restricted to underlying-cause mortality and therefore does not capture all deaths in which cardiomyopathy contributed. Individual-level information on alcohol consumption, abstinence, ventricular function, comorbidities, heart failure therapies, alcohol use disorder treatment, socioeconomic factors, and healthcare access was unavailable. Some death counts required for the aggregated age-specific analyses were suppressed in CDC WONDER and therefore could not be directly ascertained. These suppressed counts were coded as zero for aggregation, which may have resulted in conservative underestimation of CDRs in low-event strata. Changes in racial classification limit direct comparisons across some racial categories between periods, and pooled state estimates do not establish continuous state-specific temporal trends. Finally, this was a descriptive ecological analysis without formal temporal trend, between-period, or between-group hypothesis testing; therefore, statistical significance and causality cannot be inferred.

## Conclusions

Mortality from several major cardiomyopathy subtypes declined substantially in the United States between 1999 and 2024, whereas ACM showed a smaller long-term reduction and an increase during the early 2020s. ACM mortality remained disproportionately concentrated among males, particularly middle-aged and older males, with additional racial and geographic disparities. These findings identify ACM as a potentially modifiable cardiovascular burden and support closer integration of cardiovascular prevention and heart failure care with systematic assessment of alcohol exposure and evidence-based treatment of alcohol use disorder.
